# Cognitive Resilience to Psychological Stress in Military Personnel

**DOI:** 10.3389/fpsyg.2022.809003

**Published:** 2022-03-16

**Authors:** Andrew Flood, Richard J. Keegan

**Affiliations:** ^1^University of Canberra Research Institute for Sport and Exercise, University of Canberra, Bruce, ACT, Australia; ^2^Discipline of Psychology, Faculty of Health, University of Canberra, Bruce, ACT, Australia; ^3^Discipline of Sport and Exercise Science, Faculty of Health, University of Canberra, Bruce, ACT, Australia

**Keywords:** mental fatigue, pressure, mental health, cognitive performance, stress

## Abstract

Military personnel often perform complex cognitive operations under unique conditions of intense stress. This requirement to perform diverse physical and mental tasks under stress, often with high stakes, has led to recognition of the term ‘tactical athlete’ for these performers. Impaired cognitive performance as a result of this stress may have serious implications for the success of military operations and the well-being of military service men and women, particularly in combat scenarios. Therefore, understanding the nature of the stress experienced by military personnel and the resilience of cognitive functioning to this stress is of great importance. This review synthesises the current state of the literature regarding cognitive resilience to psychological stress in tactical athletes. The experience of psychological stress in military personnel is considered through the lens of the Transactional Theory of stress, while offering contemporary updates and new insights. Models of the effects of stress on cognitive performance are then reviewed to highlight the complexity of this interaction before considering recent advancements in the preparation of military personnel for the enhancement of cognitive resilience. Several areas for future research are identified throughout the review, emphasising the need for the wider use of self-report measures and mixed methods approaches to better reflect the subjective experience of stress and its impact on the performance of cognitive operations.

## Introduction

Stress and its impact on a range of cognitive processes continues to be a subject of intense scientific investigation. Ongoing research has led to the emergence of the concept of *cognitive resilience*, explaining the degree to which cognitive functions can withstand, or be resilient to, the effects of stress (Staal et al., 2008). In military personnel, cognitively demanding tasks are regularly performed under stress, with a survey conducted by the U.S. Department of Defence finding that 87% of military personnel report experiencing at least some stress as a result of their work (Bray et al., 2009). To denote these demands, the term *tactical athletes* was reportedly offered by the former chief of staff of the US Army (Hammermeister et al., 2012). Because of the prevalence of stress and the fact that the performance of cognitive tasks often carries significant consequences, ensuring and promoting cognitive resilience in military personnel is a high priority. Indeed, the development of mental skills training programs in military settings (Cohn et al., 2010; Jha et al., 2017a) is, in part, a recognition of the importance of maintaining cognitive performance under stress. Importantly, cognitive resources are required for self-regulation of effort, attention and emotional control, with real implications for the management of daily living demands and mental health (Martin et al., 2019; Rabheru et al., 2021; Rezapour et al., 2021).

In a previous review, we have demonstrated that military personnel are faced with a range of environmental stressors and that these stressors, including heat, cold and altitude, can have consequences for cognitive processes, including attention and working memory (Martin et al., 2019). We have also synthesised the state-of-the-art evidence linking cognitive performance to physiological variations such as physical fatigue, sleep deprivation, nutrition and aerobic fitness (Martin et al., 2020). This narrative review aims to extend upon this work by outlining the role of psychological factors in cognitive resilience. In the following text we review the concept of psychological stress and highlight how psychological processes of cognitive appraisal and coping can act to mitigate the effects of environmental stressors on cognitive performance in military personnel. While the effects of stress on cognition and operational performance have been the subject of several previous reviews (see Staal, 2004; Kavanagh, 2005; Driskell et al., 2006; Lukey and Tepe, 2008), the present paper is intended to provide an updated and targeted examination of the effects of psychological stress on cognition within a military context. Recent developments in the enhancement of cognitive resilience are also considered against existing and well-established theoretical models, in an attempt to highlight gaps in knowledge and areas for future investigation.

## Cognitive Resilience: Applications and Theoretical Explanations

Although an all-encompassing definition remains elusive, in its use in the behavioural sciences, resilience is typically thought to involve two components: adversity and positive adaptation (Luthar and Cicchetti, 2000). Accordingly, Luthar and Cicchetti (2000, p. 858) define resilience as a process of displaying ‘positive adaptation despite experiences of significant adversity or trauma’. Resilience can also be framed as a trait, denoting certain personal attributes allowing an individual to positively adapt to demands (Fletcher and Sarkar, 2012). In military settings, Mastroianni et al. (2008, p. 43) draw heavily from Lazarus and Folkman’s (1984) Transactional Theory of stress (see “Psychological Stress” below), defining resilience as ‘the interaction between individuals and their environment that leads to the achievement and maintenance of effective health and performance under stress’. In all cases, this interest in resilience as a personal strength represents a polar shift away from examining risk factors associated with problematic or dysfunctional outcomes (Rutter, 1987; Fergus and Zimmerman, 2005). The concept of cognitive resilience has followed from this literature to describe the specific effects of stress on cognitive functioning. Cognitive resilience has been defined by Staal et al. (2008, p. 260) as the ‘capacity to overcome the negative effects of setbacks and associated stress on cognitive function or performance’. This definition maintains the core characteristics of psychological resilience in adversity - or in this case, stress - and positive adaptation.

There is a high degree of interest in cognitive resilience within the scientific literature, studying the resilience of a wide range of cognitive processes against the effects of stress in various populations. For example, Mujica-Parodi et al. (2008) examined the impact of body fat percentage on cognitive resilience, finding that those with higher body fat were less resilient to the effects of a real-world stressor (skydiving) on spatial processing, attention and working memory. In developmental neuropsychology, cognitive resilience has been used to explain individual differences in age-related declines in cognitive capacity (Yaffe et al., 2009) and the development of cognitive impairment in Alzheimer’s Disease (Arnold et al., 2013; Negash et al., 2013). In athletes, sustained attention, as assessed in a Stroop task, has been shown to be resilient to high levels of stress resulting from physical and academic demands (Shields et al., 2017). The concept of cognitive resilience has also been applied to assess the effects of the unique stressors experienced by military personnel on cognitive functioning (Morgan et al., 2002, 2006; Hansen et al., 2009; Taverniers et al., 2011). Cognitive resilience is, therefore, important in many settings. In military settings populated by ‘tactical athletes’, successful cognitive performance under stress carries significant consequences. Before delving deeper, however, an issue that is inherent in the examination and theoretical explanation of cognitive resilience is the definition of what constitutes stress. Below, we highlight the complexity of the concept of psychological stress before reviewing situations where cognitive resilience to psychological stress is challenged in military personnel.

## Stress

Historically, public and scientific interest in the concept of stress was borne out of what is now viewed as the stressors of war, particularly World War II (Lazarus, 2007). Grinker and Spiegel (1945) wrote of the stress of war, with a focus on Air Force pilots. Military organisations were concerned with understanding the effects of stress on the performance of military personnel in battle, with the intention of using this information to inform the recruitment of those best able to maintain performance under stress (Grinker and Spiegel, 1945; Lazarus and Folkman, 1984). In many ways, very little has changed, as evidenced by the military interest in the related concept of cognitive resilience described above. Importantly, however, the concept of stress permeated beyond the military setting and stress was recognised as relevant to the lives of civilians. For a comprehensive reflection on the history of stress research, we direct interested readers to *Stress and Coping* (Lazarus, 2007). Below, we define psychological stress, drawing on the transactional theory of stress and coping, in order to contextualise the subsequent discussion of the effects of stress on cognition in military personnel.

### Psychological Stress

The Transactional Theory of Lazarus and colleagues defined psychological stress as the ‘relationship between the person and the environment that is appraised by the person as taxing or exceeding his or her resources and endangering his or her well-being’ (Lazarus and Folkman, 1984, p. 21). Stress, therefore, involved a subjective component that mediated the stressor-response relationship (Lazarus and Folkman, 1984). This definition emphasised the individual differences in responses to a stressor, proposing that one’s degree of vulnerability to stress was due to the ‘cognitive processes that intervene between the encounter and the reaction’ (Lazarus and Folkman, 1984, p. 23). Appraisal and coping were identified as the two cognitive processes mediating this person-environment transaction.

Appraisal is a cognitive process of evaluating the relevance of the person-environment transaction to one’s well-being (Folkman et al., 1986a). According to Lazarus and Folkman (1984), appraisal occurs in two main forms. Primary appraisal is the evaluation of a person’s ‘stake’ in the person-environment transaction. This primary appraisal can be further classified into three forms. An *irrelevant* appraisal results from a transaction that is not deemed to be threatening. A *benign-positive* appraisal results from a person-environment transaction that is perceived as positive or expected to have a positive impact on well-being. Finally, *stress* appraisal results from a person-environment transaction that is perceived as negative or is expected to have a negative impact on well-being. Stress appraisal itself has three forms: harm, threat and challenge (Lazarus and Folkman, 1984; Folkman et al., 1986a). Briefly, harm appraisals relate to stressors that have already damaged an individual’s well-being, while threat appraisals relate to the anticipation of harm. Challenge appraisal results from a person-environment transaction that has the potential to promote personal growth after a degree of personal difficulty or challenge. Secondary appraisal involves the active evaluation of one’s capacity to manage the transaction, including the consideration of available coping resources (Lazarus and Folkman, 1984). Importantly, as Lazarus and Folkman (1984) highlight, the use of ‘primary’ and ‘secondary’ is not intended to denote a temporal relationship, nor degree of importance for either appraisal process. Indeed, each category of appraisal interacts to produce the degree of stress experienced by the individual and the coping approach utilised for any given transaction (Lazarus and Folkman, 1987).

Following the appraisal of the person-environment transaction, coping resources are mobilised to allow the individual to cope with the resulting stress. According to the Transactional Theory, coping is the ‘constantly changing cognitive and behavioural efforts to manage specific external and/or internal demands that are appraised as taxing or exceeding the person’s resources’ (Lazarus and Folkman, 1984, p. 141). What is emphasised in this definition is that coping – at least in its use in the Transactional Theory – is not an automated response to a stressor but rather an effortful process that evolves to reflect the changing nature of the person-environment encounter (Lazarus and Folkman, 1984; Folkman et al., 1986a; Lazarus, 2000).

The original Transactional Theory outlined two ways of coping: emotion- and problem-focussed coping (Lazarus and Folkman, 1984). Emotion-focussed coping refers to coping strategies intended to regulate the emotional responses to the stressor, while problem-focussed coping strategies are used with the intention of impacting on or altering the stressor itself (Lazarus and Folkman, 1984). However, Folkman (1997) later added a third category of coping, meaning-focussed coping. When stressors persist despite the activation of problem- and emotion-focussed coping, meaning-focussed coping strategies are initiated (Folkman, 2008). This involves the use of beliefs, values and existential goals to find meaning in stressful encounters and to sustain coping efforts (Folkman, 2008). This addition may prove to be particularly relevant to the management of stress in military settings, as discussed below.

The two cognitive processes of coping and appraisal are thought to interact in a number of ways. First, the appraisal of the person-environment transaction can impact on the use and effectiveness of coping approaches (Baum et al., 1983; Folkman, 1984; Folkman et al., 1986b; Lazarus and Folkman, 1987; Folkman and Moskowitz, 2000; Roesch et al., 2002; Nicholls et al., 2014, 2016). For example, problem-focussed coping is more likely to be used and be successful if the person-environment transaction is appraised (secondary-appraisal) as within one’s control. Nonetheless, in cases where altering the transaction is difficult or impossible, emotion-focussed coping is more likely to be used. Appraisal and coping also interact through a process of reappraisal. Following coping efforts, a reappraisal of the changing person-environment transaction occurs (Lazarus and Folkman, 1984), adjusting the perceived stress and the coping strategy used. In summary then, the Transactional Theory suggests that the appraisal, coping and reappraisal of the person-environment transaction mediates the intensity of stress that is perceived. Importantly, even at its inception, Lazarus and Folkman’s (1984) Transactional Theory proposed a dynamic and adaptive process; and this aligns well with the literature on cognitive resilience.

Despite the wide-spread adoption of the Transactional Theory in stress and coping research, numerous other categorisations of coping strategies have been formulated. For example, similar to the addition of meaning-based coping by Folkman (1997), Billings and Moos (1982) added appraisal-focussed coping to emotion- and problem-focussed approaches. Approach and avoidance coping strategies, defined as ‘cognitive and emotional activity that is oriented either toward [approach] or away [avoidance] from threat’ (Roth and Cohen, 1986, p. 813), have also been widely investigated. Skinner et al. (2003) conducted a comprehensive review into the categorisation of coping strategies, which identified literature on 400 coping strategies, leading to criticism of both the emotion-focussed vs. problem-focussed and approach vs. avoidance classifications. They argued instead for a categorisation based on ‘action types’, outlining 12 families of action types that correspond to challenges and threats to relatedness, competence and autonomy. Distinct ‘root action tendencies’ align with those families identified in this hierarchical model and include, for example, support seeking, problem solving, escape and submission. Each family of root action tendencies then distill down to lower-order coping approaches such as help seeking, strategizing, procrastination and self-blame. This hierarchical model is now widely cited and has been applied in military settings (Rossetto, 2015).

Upon reviewing this research on psychological stress, we therefore highlight the importance of subjective perception in the experience of stress, arguing that a stressor causes stress only if is perceived as stressful (Roesch et al., 2002). By outlining the role of cognitive appraisal, coping and reappraisal, this literature has expanded our understanding of the individual differences in the stress experienced as a result of a perceived or anticipated stressor. This understanding of stress provides a context through which we can begin to understand cognitive resilience as defined by Staal et al. (2008; see above). We extend upon this discussion of the nature of stress and its impacts on cognition below, by discussing the psychological stress experienced in military settings before addressing the impact that this has on cognitive performance.

## Psychological Stress in the Military

Significant efforts have been made to profile the types of stressors that military personnel are exposed to. These efforts have identified a wide range of both military-specific and non-military-specific stressors that military personnel must overcome, or be resilient to, in order to maintain psychological and cognitive functioning. After an extensive investigation of the nature of the stressors experienced in military operations, Bartone et al. (1998) identified five overarching dimensions of stress reported by U.S. military personnel. These included *isolation, ambiguity, powerlessness, boredom* and *danger*. Later, Bartone (2006) added a sixth dimension of *workload* to account for the increasing demands placed on military personnel in the form of longer working hours and increased frequency of deployments. Encompassed within these six dimensions are a range of specific stressors reported by military personnel, including separation from family and friends (*isolation*), the fluid nature of the mission (*ambiguity*), an inability to influence changes occurring back home (*powerlessness*), repetitive work (*boredom*), the risk of injury or death (*danger*) and the high frequency of deployment (*workload*; see Bartone, 2006 for comprehensive review). We note here that the stressors identified were collected using self-report measures. As a result, they represent stressors that have been appraised as stressful, aligning with the definition of psychological stress provided by the Transactional Theory.

From the dimensions identified in such analyses, much of the stress experienced by military personnel has parallels with the stressful experiences of civilian populations. For example, stress relating to workload extends well-beyond military settings, with workload considered a major contributor to occupational stress and burnout (Jex, 1998). Similarly, boredom is a common complaint across a range of occupational domains (Fisherl, 1993). Research in non-deployed military personnel certainly supports this argument, with work-related stressors such as changes in responsibilities, staffing and work hours being the most commonly reported sources of stress (Pflanz, 2001; Pflanz and Sonnek, 2002; Pflanz and Ogle, 2006). It is clear from these findings that interventions aiming to reduce stress and its impact on the cognitive functioning of military personnel should not disregard the prevalence of these common occupational stressors.

However, unique, military-specific stressors are also well recognised. These stressors often come in the form of combat stress, i.e., the particular requirement to take actions that may end another human’s life, or indeed the risk of losing one’s own life. As such, combat stress may result from a range of stressors, including exposure to life threatening events or the injury and death of others (Dekel et al., 2003; Hoge et al., 2004). In a survey of U.S. army personnel deployed to Iraq, almost all report being shot at (93%) or seeing dead bodies or human remains (95%). However, reported exposure to these stressors was significantly reduced in those deployed to Afghanistan (Hoge et al., 2004). Similar findings have been reported in an Australian military sample, with the threat of injury or death, seeing dead bodies, the death of a friend or co-worker, and causing death or injury to others, all listed as potentially traumatic events experienced by Australian military personnel on peacekeeping missions (Hawthorne et al., 2014). At this time, more work is needed to extend beyond simply cataloguing the combat stressors faced by military personal by assessing the appraisal of these stressors. In order to collect such data, validated measurement tools of appraisal and coping (e.g., Mikulincer and Florian, 1995) alongside qualitative interviews conducted during operational debriefing, may provide an appropriate mixed-methods approach. It is likely that this approach will clarify whether these reported combat stressors, or potentially traumatic events, are appraised as stressful, an important distinction according to the Transactional Theory of psychological stress.

The evolving nature of modern military operations presents as a challenge to profiling the types of stressors that cause psychological stress in military personnel. In their outline of the stressors of modern war, Mastroianni et al. (2008) discussed the evolving environment within which military personnel now operate and how this impacts on the types of stressors that are experienced. In particular, they highlighted the shift from traditional warfare to the constant threat of unpredictable insurgent attack in current operations in Iraq. This method of warfare is thought to remove the traditional concept of a ‘front-line’, leaving military personnel under constant threat of attack (Mastroianni et al., 2008), also referred to as being in a state of ‘persistent conflict’ (Casey, 2011). The health implications of this chronic psychological stress are perhaps most clearly represented by the high rates of post-traumatic stress disorder in military personnel returning from Iraq and Afghanistan (Hoge et al., 2004). Whether similar deleterious effects are seen in cognitive functioning during deployment or, indeed, afterwards. Are yet to be determined, but there is accumulating evidence that – in the wider population - sufferers of PTSD do tend to also exhibit memory and attention deficits, associated with changes in functional brain activity (Hayes et al., 2012).

Advancements in technology have also produced significant changes in modern military combat. The increased use of unmanned aerial vehicles (UAVs), in particular, represent this advancement in military technology (Bone and Bolkcom, 2003). Although the use of UAVs removes the threat of physical harm to the pilot, recent research suggests that UAV pilots experience high levels of psychological stress (Fitzsimmons and Sangha, 2010; Chappelle et al., 2014; Armour and Ross, 2017; Chapa, 2017). In their detailed description of the experiences of UAV pilots, Fitzsimmons and Sangha (2010) highlighted the psychological closeness that is developed between the UAV pilot and their target – for example during extended observation of daily movements - and how this closeness may account for the psychological stress that operatives experience. The physical separation from the battlefield also presents as an issue for the psychological stress experienced by UAV pilots, with many commuting to a military base from their homes each day (Armour and Ross, 2017). This leaves little time for the pilots to ‘decompress’ and make the difficult mental transition from the warzone to civilian life (Fitzsimmons and Sangha, 2010). These factors, and likely many more, emphasise the importance of continuing the investigation into the psychological stress of modern warfare and its impact on cognitive performance. However, technological advancement, such as virtual reality, also offers an opportunity to combat the effects of stress on performance, a point discussed later in this review. The close intertwining of stress and cognitive performance is exemplified in the UAV concept, perhaps particularly because the physical demands, and physical threat are removed, and yet combat stress remains closely linked with, and dependent on, cognitive performance. Developments such as the proliferation of UAV warfare help to explain the renewed emphasis on cognitive resilience in tactical athletes.

## Cognitive Resilience to Psychological Stress in the Military

As outlined above, military personnel experience a range of both military-specific and non-military-specific psychological stress. Similarly, certain cognitive functions are of particular importance in military contexts. Investigations of cognition in military personnel have adopted a range of neurocognitive tools assessing memory, visuospatial integration, reaction time and executive functions (Morgan et al., 2006, 2011; Orantes-Gonzalez and Heredia-Jimenez, 2021). These cognitive domains are thought to be important for performance within a military context, for example, in navigating unfamiliar territory, executing orders while resisting distraction or reacting to unexpected threats. Indeed, the ongoing modernisation of warfare places additional demands on soldiers’ capacity to monitor and respond to multiple sources of information (Kerick and Allender, 2004; Spivak et al., 2019; Bequette et al., 2020). Executive functions of inhibition, shifting and updating appear to be especially and increasingly relevant to the military context (Blacker et al., 2019) and efforts to improve executive functioning translate to improved performance in simulated, military-relevant tasks (Biggs et al., 2015). However, it should be noted that military personnel perform diverse tasks that engage a range of cognitive domains (Blacker et al., 2019), such that what is essential in one role (e.g., artillery operator) may not apply in another (e.g., air traffic controller). Examinations of cognitive resilience, then, should adopt a tailored approach that consider the cognitive challenges and stressors specific to individual roles.

In investigating the effects of stress on these military-relevant cognitive functions, researchers are faced with the challenge of inducing stress in ecologically valid ways. One approach has been to use stress inoculation training methods, also referred to as sustained operations (SUSOPS) training (Vrijkotte et al., 2016). The field phase of Survival, Evasion, Resistance, and Escape (SERE) training (Doran et al., 2012), which involves subjecting military personnel to a mock prisoner of war interrogation, has been shown to impair reaction time, attention, vigilance, memory and reasoning (Lieberman et al., 2005a, 2016; Morgan et al., 2006). In another assessment of the effects of SERE training, Harris et al. (2005) examined stress-induced changes across multiple cognitive domains including reaction time, working memory and reasoning. Interestingly, only simple reaction time was shown to be impaired following SERE training, while either no change or improved performance was observed in more complex cognitive tasks such as spatial processing. It is suggested that the allocation of effort towards these more complex cognitive operations can temporarily mask the deleterious effects of stress (Harris et al., 2005). This argument for the adaptive and protective function played by the allocation of resources will be compared to alternative theoretical explanations below (see “Psychological Stress and Cognitive Performance: Theoretical Explanations”) and is central to the ongoing development of conceptual frameworks for cognitive resilience and mental fatigue in athletes (Martin et al., 2015; Filipas et al., 2020).

Beyond SERE training, Lieberman et al. (2005b) assessed the effects of U.S. Navy Seal training on cognitive performance. They report impaired reaction time, vigilance, attention and memory during the particularly intense ‘Hell Week’ portion of the program, which involves severe sleep deprivation and exposure to environmental, psychological and physical stressors (Lieberman et al., 2002). Similar stress-induced impairments in cognitive performance have been reported in paratrooper training (Sharma et al., 1994; Taverniers et al., 2011) and other stress inoculation simulations (Taverniers et al., 2010, 2013). As these training environments are designed to mimic many of the characteristics of military operations, such as sleep loss, physical discomfort, perceived threat and intense physical activity, the reported decrements in cognitive performance may be representative of the expected changes occurring in active military operations.

An important limitation of the research presented above, however, is the lack of subjective measurement of stress. Therefore, although some research suggests that military training environments and simulations induce psychological stress (Kreuz et al., 1972; Morgan et al., 2000), the degree to which changes in cognitive functioning are due to the psychological appraisal of these situations remains largely unclear. Attempts have been made to overcome this issue by reporting on changes in subjective perceptions of task load (Taverniers et al., 2010, 2011) and mood (Harris et al., 2005; Lieberman et al., 2005b) that occur during training simulations. However, these measures do not directly assess psychological stress as defined by the Transactional Theory of stress (Lazarus and Folkman, 1984). Future research should use established measures of both state and trait perceived stress, such as visual analogue scales (Hellhammer and Schubert, 2012) and the Perceived Stress Scale (Cohen et al., 1983), to uncover the degree to which these training simulations induce psychological stress and whether this psychological stress impacts on cognitive performance. The inclusion of both subjective and objective measures of stress may help refine the protocols used for military training operations to better reflect and combat the apparent impact of subjective stress appraisal on cognitive functioning.

## Psychological Stress and Cognitive Performance: Theoretical Explanations

Despite numerous attempts, the development of a comprehensive theoretical explanation of the effects of stress on cognition, has proven difficult. This difficulty is due to the complexity of *both* stress and cognition. For example, the source of stress and its intensity, controllability and duration have all been shown to influence the changes observed in cognitive functioning (Sandi, 2013). The characteristics of the specific cognitive operation under investigation also influences the degree of resilience to stress (Sandi, 2013). Theoretical explanations of cognitive resilience must account for the range of possible consequences resulting from these interactions between stress-related and cognition-related factors. It is beyond the scope of this review to fully account for the number of theories that have been presented to explain the effects of stress on cognition. Instead, in the following text we highlight a sample of the theoretical explanations that have been widely adopted, particularly in military psychology. It is not our intention here to argue for one particular theoretical position. Rather, we aim to identify common themes that permeate across theories, in order to provide a framework through which to consider the findings presented above regarding the extent to which cognitive functioning in military personnel can be made resilient to psychological stress.

### Maximal Adaptability Model

Hancock and Warm (1989) provide a model of the effects of stress on cognitive performance. This model, referred to as the Maximal Adaptability Model acknowledged that the cognitive task itself is a primary source of stress. In their dynamic model, Hancock and Warm (1989) argued that psychological and physiological adaptive mechanisms act to buffer the effects of stress on performance. Here, psychological adaptation refers to the allocation of attentional resources. This borrowed heavily from Kahneman’s (1973) theory of attention and effort allocation, where attention was presented as a limited, depletable resource. Physiological adaptation refers to homeostatic regulatory functions that attempt to accommodate the effects of stressors (Hancock and Warm, 1989).

The Maximal Adaptability Model predicts that when stressors are minor, psychological and physiological adaptations can effectively buffer any disruptions to performance. However, as stressors progress to the extremes of hyper- or hypo-stress, limits of maximal psychological and physiological adaptability may be exceeded, resulting in dynamic instability. Overall, Hancock and Warm’s (1989) dynamic model suggests that hyper- and hypo-stress impacts on cognitive performance by depleting attentional resources and overwhelming homeostatic control systems. Given the high intensity and extended duration of the stressors associated with the military simulations described above, it is likely that limits of psychological and physiological maximal adaptability were exceeded. This would account for the observed impairments in cognitive functioning. Therefore, the dynamic model of Hancock and Warm (1989) may (still) serve as a theoretical explanation for the effects of stress on cognition in military settings. However, while physiological responses to stress inoculation training have been examined (Taverniers et al., 2010, 2013; Taylor and Schatz, 2011), the deployment and depletion of attentional resources (psychological adaptation) during training simulations, and indeed during active combat, require examination.

### Compensatory Control Model

A similar explanation of the impact of stress on performance is presented in Hockey’s (1997) Compensatory Control Model. Two levels of control are proposed in this model. In well-learned tasks under conditions of low stress, performance is maintained by an automatic system of control that does not tax limited energetic resources. When task demands are registered by an ‘effort monitor’ as exceeding the capacity of this lower automatic system, a higher supervisory controller is activated to initiate a compensatory control response. This response may involve the mobilisation of effort to protect task performance. However, much like attention in the work of Hancock and Warm (1989), the Compensatory Control Model considers effort a limited resource (Hockey, 1997). Therefore, while effort allocation may effectively, but temporarily, maintain primary task performance, prolonged or particularly intense stress may deplete resources to the point where performance decrements are observed (Hockey, 1997), as seen in the cognitive impairment resulting from military simulations. Additionally, Hockey (1997) suggested that so-called ‘latent decrements’ may occur outside of the primary task. For example, peripheral task performance may be impaired through attentional tunnelling (Kohn, 1954; Staal, 2004) and the use of less effortful cognitive strategies, such as heuristics (Gigerenzer and Selten, 2001), in non-primary tasks. In military settings, changes in mood (Harris et al., 2005; Lieberman et al., 2005a) or breakdowns in teamwork (Driskell et al., 1999) may represent latent decrements resulting from the allocation of effort to maintain primary task performance.

According to the Compensatory Control Model, however, the allocation of effort is only one protection strategy that may be initiated by the supervisory controller. A second strategy, which avoids the aversive and costly mobilisation of effort, is to instead adjust performance targets (Hockey, 1997). Although this passive coping strategy maintains energetic resources, task performance is impaired. Indeed, passive coping may, and often does, manifest as complete task disengagement (Hockey, 1997). Given the often fixed and externally imposed nature of the performance targets in military combat settings, it is unclear whether passive coping strategies are possible for military personnel. Therefore, it is likely that in the stress inoculation training described above, and indeed during active combat, effort allocation may be the only protective strategy available to the supervisory controller. We encourage future research to consider whether, under the stress of combat, military personnel select to adjust their performance targets or instead sacrifice secondary tasks by allocating effort to primary targets.

### Attentional Control Theory

Furthering the emphasis on the protective reallocation of attentional reserves is the Attentional Control Theory (Eysenck et al., 2007). Attentional Control Theory extends on Eysenck and Calvo’s (1992) processing efficiency theory which considered the effects of stress and anxiety on cognition in terms of effectiveness and efficiency. It was argued that anxiety creates a state of self-preoccupation, drawing from limited attentional resources, which forces higher levels of effort to be allocated to maintain task performance. This increased allocation of effort may preserve performance quality (effectiveness), but it does so at the expense of decreased processing efficiency. While Attentional Control Theory maintains this distinction between processing efficiency and performance effectiveness, it describes several important extensions (Eysenck et al., 2007). Specifically, Attentional Control Theory provides a more nuanced explanation of attentional demands, suggesting that decrements in processing efficiency are due to an anxiety-induced shift in attention away from the pursuit of goals and towards salient stimuli. Further, according to Attentional Control Theory, anxiety is most likely to affect processing efficiency in tasks requiring the executive functions of inhibition and shifting, since these functions ensure attention is directed toward task-relevant stimuli. As described above, these cognitive operations are particularly relevant to a military context.

Attentional Control Theory has been used to explain cognitive deficits resulting from anxiety and stress across a range of settings. For example, supporting the theory’s predictions, those with anxiety disorders display cognitive deficits that appear to relate to reduced attentional capacity (Stefanopoulou et al., 2014). In a military context, Attentional Control Theory’s description of differential effects of anxiety across executive functions has been used to guide the development of cognitive training interventions that target those executive functions (inhibition and shifting) most threatened by anxiety (Ben-Avraham et al., 2021). Attentional Control Theory can also be used to explain decrements in shooting performance and increases in effort in simulated military operations designed to provoke anxiety (Nibbeling et al., 2014). Such findings highlight the potential applications and practical utility of the predictions of Attentional Control Theory for tactical athletes in military settings.

### Summary and Critique

Common across all theories described above is the effortful allocation and reallocation of attention. This is thought to buffer the effects of stress on cognitive performance (task effectiveness), underpinning the conceptualisation of cognitive resilience. However, in monitoring cognitive resilience, measures should extend beyond task performance to also consider processing efficiency. This can be achieved by measuring subjective workload or perceived effort to determine the potential that effort allocation is protecting performance from the effects of stress. Uncovering regular, compensatory allocation of effort to sustain performance under stress may help to (1) detect potential threats to cognition before performance is degraded and (2) avoid cognitive and emotional burnout in military personnel.

## Enhancing Cognitive Resilience

An understanding of the psychological stress experienced in military personnel and the impact of this stress on cognitive functioning offers avenues for enhancing cognitive resilience. However, the limits of cognitive resilience are bounded by two key considerations. First, stress is an inescapable and, therefore, inevitable part of life (Lazarus and Folkman, 1984). Second, cognitive performance is rarely immune to the effects of stress (Kavanagh, 2005). Despite these constraints, cognitive resilience can be enhanced (see below) and individual differences do exist, both in the psychological stress response (Parkes, 1986) and cognitive resilience to stress (Staal et al., 2008). Identifying the characteristics that explain these individual differences has clear applications in military settings, particularly in the selection of cognitively resilient military personnel and the determination of ‘cognitive readiness’ (Grier, 2012). Indeed, the Transactional Theory and Maximal Adaptability Model have been used to develop comprehensive assessment tools, such as the Readiness Assessment and Monitoring System (Cosenzo et al., 2007), that aim to predict cognitive performance under stress in military settings.

The capacity to train or enhance cognitive resilience also has obvious practical implications in military settings. A review by Kavanagh (2005) considered two points of moderation, where various factors can intervene in the effects of stress on performance. The first point of intervention (type 1 moderators), includes factors that moderate the stress that results from the presentation of a stressor (Kavanagh, 2005). While Kavanagh (2005) was concerned with physiological responses to stressors, this first point of intervention also applies in psychological stress. Specifically, type 1 moderators can be seen as equivalent to the person-environment transaction described by Lazarus and Folkman (1984). The second point of intervention (type 2 moderators) proposed in Kavanagh’s (2005) model, includes factors that moderate the effects of stress on performance. That is, once stress is experienced, what are the factors that moderate its effects on performance? Despite the focus on the physiological stress response, this two-point moderation model provides a useful heuristic for the consideration of interventions to enhance cognitive resilience in the military.

Several methods of enhancing cognitive resilience in military personnel were reviewed by Staal et al. (2008), including the use of phased training techniques (see Keinan et al., 1990 for explanation). However, since that review, considerable gains have been made in the development of programs that aim to enhance cognitive resilience in military personnel. Mindfulness-based interventions, in particular, have proven to be especially effective in enhancing cognitive resilience. For example, Jha and colleagues (Jha et al., 2010, 2015, 2017a,b) report that while decays in working memory and attentional capacity occur in military personnel during the highly stressful pre-deployment phase, those engaging in mindfulness-based practice as part of an 8-week training program appear to be resilient to these effects, with some even displaying improvements in attention and working memory. This intervention can be seen as acting on both points of moderation described in Kavanagh’s (2005) model. Mindfulness interventions can act to reduce the psychological stress response (Baer et al., 2012), thereby presenting as a type 1 moderator. In fact, Johnson et al. (2014) reported decreased psychological stress responses to stress-inoculation training scenarios in military personnel after an 8-week mindfulness intervention. As a type 2 moderator, mindfulness interventions have been shown to improve performance in a range of cognitive operations (Jha et al., 2007; Zeidan et al., 2010) and may, therefore, protect or enhance cognitive reserves that are threatened by stress. Physical training, particularly in tasks requiring the regulation of effort and pacing (i.e., endurance) has also shown promise as a way of building cognitive resilience to the effects of mental fatigue (Filipas et al., 2020).

Similarly, Virtual Reality (VR) technology has been used in military training environments to train cognitive resilience to stress. Most commonly, VR technology has been paired with cognitive-behavioural therapy as a tool to gradually and safely expose military personnel suffering from PTSD to anxiety provoking stimuli so that therapeutic cognitive reorientation can take place (Rizzo et al., 2011; Seitz et al., 2014). However, VR technology has also been ‘retooled’ for use during pre-deployment training. Adopting the principles of stress inoculation training programs outlined above (see also Meichenbaum, 1977), VR technology has been used to present stress-inducing virtual scenarios to encourage adaptive responses in military personnel (Pallavicini et al., 2016; Binsch et al., 2021). For example, in the ‘Stress Resilience in Virtual Environments’ (STRIVE) project, virtual environments depicting combat scenarios in Iraq and Afghanistan are used to facilitate adaptive coping and train cognitive appraisal processes to be oriented toward challenge, rather than threat, appraisals (Wiederhold and Wiederhold, 2008; Buckwalter et al., 2012). This technology has also allowed for more realistic and therefore, ecologically valid tools for assessing cognitive operations (Parsons and Rizzo, 2008) and has been shown to enhance military operational performance (Wiederhold and Wiederhold, 2008). Therefore, by acting on stress appraisal and reducing the impact of stress on performance, VR-based stress inoculation training can influence cognitive resilience at both points of moderation proposed by Kavanagh (2005). With such wide-ranging applications, VR presents as an exciting opportunity to enhance the psychological and cognitive readiness of military personnel. However, while physiological stress markers have been routinely monitored during VR-based stress inoculation training (Rizzo et al., 2013), more work is needed to assess the impact on perceived stress during the inoculation training and in real-world scenarios.

It has recently been suggested that a return to past approaches is needed to combat the risk-averse nature of current military training protocols (Nindl et al., 2018). While an overly risk-averse direction may ultimately reduce the combat readiness of military personnel, continual refinement of training protocols is necessary to acknowledge the changing nature of the stressors faced by military personnel and the many advancements made in deployment preparation. Promising areas of research into the psychological preparation of military personnel for modern combat are wide-ranging, certainly extending beyond the examples provided here. Given its importance in military operations, further exploration of methods to promote cognitive resilience, grounded in the theoretical models outlined above, is encouraged. In the modern context, online and app-based interventions may also be adopted to support the monitoring of stressful stimuli, changes to stress appraisal, and also optimal coping strategies, with a view to shifting the scientific support closer towards the moments and locations where the stress is experienced.

## Conclusion

The stressors faced by military personnel are diverse, ranging from boredom to the threat of injury and death. Advancements in technology and the nature of war mean that these stressors are also constantly evolving. It is, therefore, difficult to profile the stress experienced by military personnel in modern military operations and to determine the impact of this stress on cognitive performance. However, despite the diversity and evolution of these stressors, this review highlights that existing theoretical models remain relevant to understanding cognitive resilience in military settings. The Transactional Theory emphasises the importance of appraisal and coping for the subjective experience of stress. Attentional Control Theory and the compensatory control and maximal adaptability models explain how stress may impact on cognitive functioning by threatening limited reserves of effort and attention. Importantly, these existing theoretical perspectives emphasise that stress-induced changes in cognition may not initially be detected through decrements in performance, but instead through decreased efficiency. Further incorporation of these models into military settings will provide a platform upon which to advance our understanding of cognitive resilience to psychological stress. Several areas for future investigation have been identified throughout this review. Taken together, these recommendations identify that while the stressors faced by military personnel have been well-documented, more work is needed to determine how these stressors are appraised. This will better inform our understanding of the lived, subjective experience of stress in military personnel. It is only then that we can begin to appreciate the complexity of cognitive resilience in military settings and better prepare military personnel for the realities of modern warfare.

## Author Contributions

AF led in the planning, research, and drafting of this paper. RK liaised with the key stakeholders, agreed the necessity of the review, and reviewed and edited the drafts during the development of the paper. RK and AF worked together to plan the paper at the design stage. All authors contributed to the article and approved the submitted version.

## Funding

This research was funded by a grant from Defence Science Technology, Australia, as part of the ‘Human Performance Research Network’ (‘HPRnet’ – Award ID: 2016000167), in collaboration with Australian Army. The funding source was not involved in the preparation of the article for publication.

## Conflict of Interest

The authors declare that the research was conducted in the absence of any commercial or financial relationships that could be construed as a potential conflict of interest.

## Publisher’s Note

All claims expressed in this article are solely those of the authors and do not necessarily represent those of their affiliated organizations, or those of the publisher, the editors and the reviewers. Any product that may be evaluated in this article, or claim that may be made by its manufacturer, is not guaranteed or endorsed by the publisher.

## References

[ref1] Armour C. Ross J. (2017). The health and well-being of military drone operators and intelligence analysts: a systematic review. Mil. Psychol. 29, 83–98. doi: 10.1037/mil0000149

[ref2] Arnold S. E. Louneva N. Cao K. Wang L.-S. Han L.-Y. Wolk D. A.. (2013). Cellular, synaptic, and biochemical features of resilient cognition in Alzheimer's disease. Neurobiol. Aging 34, 157–168. doi: 10.1016/j.neurobiolaging.2012.03.004, PMID: 22554416 PMC3478410

[ref3] Baer R. A. Carmody J. Hunsinger M. (2012). Weekly change in mindfulness and perceived stress in a mindfulness-based stress reduction program. J. Clin. Psychol. 68, 755–765. doi: 10.1002/jclp.21865, PMID: 22623334

[ref4] Bartone P. T. (2006). Resilience under military operational stress: can leaders influence hardiness? Mil. Psychol. 18, S131–S148. doi: 10.1207/s15327876mp1803s_10

[ref5] Bartone P. T. Adler A. B. Vaitkus M. A. (1998). Dimensions of psychological stress in peacekeeping operations. Mil. Med. 163, 587–593. doi: 10.1093/milmed/163.9.587, PMID: 9753982

[ref6] Baum A. Fleming R. Singer J. E. (1983). Coping with victimization by technological disaster. J. Soc. Issues 39, 117–138. doi: 10.1111/j.1540-4560.1983.tb00144.x

[ref7] Ben-Avraham R. Afek A. Berezin Cohen N. Davidov A. Van Vleet T. Jordan J.. (2021). Feasibility and preliminary effectiveness of mobile cognitive control training during basic combat training in the military. Mil. Psychol. 1–13. doi: 10.1080/08995605.2021.1969162 PMC10013478 38536343

[ref8] Bequette B. Norton A. Jones E. Stirling L. (2020). Physical and cognitive load effects due to a powered lower-body exoskeleton. Hum. Factors 62, 411–423. doi: 10.1177/0018720820907450, PMID: 32202434

[ref9] Biggs A. T. Cain M. S. Mitroff S. R. (2015). Cognitive training can reduce civilian casualties in a simulated shooting environment. Psychol. Sci. 26, 1164–1176. doi: 10.1177/0956797615579274, PMID: 26170262

[ref10] Billings A. C. Moos R. H. (1982). Psychosocial theory and research on depression: an integrative framework and review. Clin. Psychol. Rev. 2, 213–237. doi: 10.1016/0272-7358(82)90013-7

[ref11] Binsch O. Bottenheft C. Landman A. Roijendijk L. Vermetten E. H. G. J. M. (2021). Testing the applicability of a virtual reality simulation platform for stress training of first responders. Mil. Psychol. 33, 182–196. doi: 10.1080/08995605.2021.1897494 PMC10013210 38536243

[ref12] Blacker K. J. Hamilton J. Roush G. Pettijohn K. A. Biggs A. T. (2019). Cognitive training for military application: a review of the literature and practical guide. J. Cogn. Enhanc. 3, 30–51. doi: 10.1007/s41465-018-0076-1

[ref13] Bone E. Bolkcom C. (2003). Unmanned Aerial V ehicles: Background and Issues for Congress. Library Of Congress Washington Dc Congressional Research service.

[ref14] Bray R. M. Hourani L. L. Olmsted K. L. Witt M. Brown J. M. Pemberton M. R.. (2009). 2005 Department of defense survey of health related behaviors among active duty military personnel: A Component of the Defense Lifestyle Assessment Program (RTI/7841/106-FR). Retrieved from Research Triangle Park, NC.

[ref15] Buckwalter J. G. Rizzo A. John B. Newman B. Williams J. Parsons T. (2012). Strive: Stress resilience in virtual environments. Paper presented at the Virtual Reality Short Papers and Posters (VRW), 2012 IEEE, Orange County, CA, USA. 22357022

[ref16] Casey G. W. Jr. (2011). Comprehensive soldier fitness: a vision for psychological resilience in the US Army. Am. Psychol. 66, 1–3. doi: 10.1037/a0021930, PMID: 21219041

[ref17] Chapa J. O. (2017). Remotely piloted aircraft, risk, and killing as sacrifice: the cost of remote warfare. J. Military Ethics 16, 256–271. doi: 10.1080/15027570.2018.1440501

[ref18] Chappelle W. L. McDonald K. D. Prince L. Goodman T. Ray-Sannerud B. N. Thompson W. (2014). Symptoms of psychological distress and post-traumatic stress disorder in United States air Force “drone” operators. Mil. Med. 179(Suppl. 8), 63–70. doi: 10.7205/MILMED-D-13-00501, PMID: 25102551

[ref19] Cohen S. Kamarck T. Mermelstein R. (1983). A global measure of perceived stress. J. Health Soc. Behav. 24, 385–396. doi: 10.2307/2136404 6668417

[ref20] Cohn A. Hodson S. Crane M. (2010). Resilience training in the Australian defence force. InPsych: the Bulletin of the Australian Psychological Society. Available at: https://psychology.org.au/publications/inpsych/2010/april/cohn

[ref21] Cosenzo K. A. Fatkin L. T. Patton D. J. (2007). Ready or not: enhancing operational effectiveness through use of readiness measures. Aviat. Space Environ. Med. 78, B96–B106. 17547310

[ref22] Dekel R. Solomon Z. Ginzburg K. Neria Y. (2003). Combat exposure, wartime performance, and long-term adjustment among combatants. Mil. Psychol. 15, 117–131. doi: 10.1207/S15327876MP1502_2

[ref23] Doran A. P. Hoyt G. Morgan C. A. (2012). “Survival, evasion, resistance, and escape (sere) training,” in Military Psychology: Clinical and Operational Applications. eds. Kennedy C. H. Zillmer E. E. (New York, NY: The Guilford Press), 306.

[ref24] Driskell J. E. Salas E. Johnston J. (1999). Does stress lead to a loss of team perspective? Group Dyn. Theory Res. Pract. 3, 291–302. doi: 10.1037/1089-2699.3.4.291

[ref25] Driskell J. E. Salas E. Johnston J. H. (2006). Decision Making and Performance Under Stress Military Life: The Psychology of Serving in Peace and Combat: Military Performance. Vol. 1. Westport, CT: Praeger Security International, 128–154.

[ref26] Eysenck M. W. Calvo M. G. (1992). Anxiety and performance: the processing efficiency theory. Cognit. Emot. 6, 409–434. doi: 10.1080/02699939208409696

[ref27] Eysenck M. W. Derakshan N. Santos R. Calvo M. G. (2007). Anxiety and cognitive performance: attentional control theory. Emotion 7, 336–353. doi: 10.1037/1528-3542.7.2.336 17516812

[ref28] Fergus S. Zimmerman M. A. (2005). Adolescent resilience: a framework for understanding healthy development in the face of risk. Annu. Rev. Public Health 26, 399–419. doi: 10.1146/annurev.publhealth.26.021304.144357, PMID: 15760295

[ref29] Filipas L. Martin K. Northey J. M. La Torre A. Keegan R. Rattray B. (2020). A 4-week endurance training program improves tolerance to mental exertion in untrained individuals. J. Sci. Med. Sport 23, 1215–1219. doi: 10.1016/j.jsams.2020.04.020, PMID: 32456979

[ref30] Fisherl C. D. (1993). Boredom at work: a neglected concept. Hum. Relat. 46, 395–417. doi: 10.1177/001872679304600305

[ref31] Fitzsimmons S. Sangha K. (2010). Killing in high definition. Technology 12, 289–292.

[ref32] Fletcher D. Sarkar M. (2012). A grounded theory of psychological resilience in Olympic champions. Psychol. Sport Exerc. 13, 669–678. doi: 10.1016/j.psychsport.2012.04.007

[ref33] Folkman S. (1984). Personal control and stress and coping processes: a theoretical analysis. J. Pers. Soc. Psychol. 46, 839–852. doi: 10.1037/0022-3514.46.4.839, PMID: 6737195

[ref34] Folkman S. (1997). Positive psychological states and coping with severe stress. Soc. Sci. Med. 45, 1207–1221. doi: 10.1016/S0277-9536(97)00040-3 9381234

[ref35] Folkman S. (2008). The case for positive emotions in the stress process. Anxiety Stress Coping 21, 3–14. doi: 10.1080/10615800701740457 18027121

[ref36] Folkman S. Lazarus R. S. Dunkel-Schetter C. DeLongis A. Gruen R. J. (1986a). Dynamics of a stressful encounter: cognitive appraisal, coping, and encounter outcomes. J. Pers. Soc. Psychol. 50, 992–1003. doi: 10.1037/0022-3514.50.5.992, PMID: 3712234

[ref37] Folkman S. Lazarus R. S. Gruen R. J. DeLongis A. (1986b). Appraisal, coping, health status, and psychological symptoms. J. Pers. Soc. Psychol. 50, 571–579. doi: 10.1037/0022-3514.50.3.571 3701593

[ref38] Folkman S. Moskowitz J. T. (2000). Positive affect and the other side of coping. Am. Psychol. 55, 647–654. doi: 10.1037/0003-066X.55.6.647, PMID: 10892207

[ref39] Gigerenzer G. Selten R. (eds.) (2001). “Rethinking rationality,” in Bounded Rationality: The Adaptive Toolbox. Vol. 1. London, England: MIT Press, 12.

[ref40] Grier R. A. (2012). Military cognitive readiness at the operational and strategic levels: a theoretical model for measurement development. J. Cogn. Engin. Dec. Making 6, 358–392. doi: 10.1177/1555343412444606

[ref41] Grinker R. R. Spiegel J. P. (1945). Men Under Stress. Philadelphia, PA: Blakiston.

[ref42] Hammermeister J. Pickering M. Lennox A. (2012). Military applications of performance psychology methods and techniques: an overview of practice and research. J. Perform. Psychol. 3, 1–12.

[ref43] Hancock P. A. Warm J. S. (1989). A dynamic model of stress and sustained attention. Hum. Factors 31, 519–537. doi: 10.1177/001872088903100503, PMID: 2625347

[ref44] Hansen A. L. Johnsen B. H. Thayer J. F. (2009). Relationship between heart rate variability and cognitive function during threat of shock. Anx. Stress Coping 22, 77–89. doi: 10.1080/10615800802272251, PMID: 18781457

[ref45] Harris W. C. Hancock P. Harris S. C. (2005). Information processing changes following extended stress. Mil. Psychol. 17, 115–128. doi: 10.1207/s15327876mp1702_4

[ref46] Hawthorne G. Korn S. Creamer M. (2014). Australian Peacekeepers: Long-Term Mental Health Status, Health Service Use, and Quality of Life - Technical Report. Australia: Department of Psychiatry, University of Melbourne.

[ref47] Hayes J. P. VanElzakker M. B. Shin L. M. (2012). Emotion and cognition interactions in PTSD: a review of neurocognitive and neuroimaging studies. Front. Integr. Neurosci. 6:89. doi: 10.3389/fnint.2012.00089 23087624 PMC3466464

[ref48] Hellhammer J. Schubert M. (2012). The physiological response to Trier social stress test relates to subjective measures of stress during but not before or after the test. Psychoneuroendocrinology 37, 119–124. doi: 10.1016/j.psyneuen.2011.05.012 21689890

[ref49] Hockey G. R. J. (1997). Compensatory control in the regulation of human performance under stress and high workload: a cognitive-energetical framework. Biol. Psychol. 45, 73–93. doi: 10.1016/S0301-0511(96)05223-4, PMID: 9083645

[ref50] Hoge C. W. Castro C. A. Messer S. C. McGurk D. Cotting D. I. Koffman R. L. (2004). Combat duty in Iraq and Afghanistan, mental health problems, and barriers to care. N. Engl. J. Med. 351, 13–22. doi: 10.1056/NEJMoa040603, PMID: 15229303

[ref51] Jex S. M. (1998). Stress and Job Performance: Theory, Research, and Implications for Managerial Practice. Thousand Oaks, CA: Sage Publications Ltd.

[ref52] Jha A. P. Krompinger J. Baime M. J. (2007). Mindfulness training modifies subsystems of attention. Cogn. Affect. Behav. Neurosci. 7, 109–119. doi: 10.3758/CABN.7.2.109, PMID: 17672382

[ref53] Jha A. P. Morrison A. B. Dainer-Best J. Parker S. Rostrup N. Stanley E. A. (2015). Minds “At attention”: mindfulness training curbs attentional lapses in military cohorts. PLoS One 10:e0116889. doi: 10.1371/journal.pone.0116889, PMID: 25671579 PMC4324839

[ref54] Jha A. P. Morrison A. B. Parker S. C. Stanley E. A. (2017a). Practice is protective: mindfulness training promotes cognitive resilience in high-stress cohorts. Mindfulness 8, 46–58. doi: 10.1007/s12671-015-0465-9

[ref55] Jha A. P. Stanley E. A. Kiyonaga A. Wong L. Gelfand L. (2010). Examining the protective effects of mindfulness training on working memory capacity and affective experience. Emotion 10, 54–64. doi: 10.1037/a0018438, PMID: 20141302

[ref56] Jha A. P. Witkin J. E. Morrison A. B. Rostrup N. Stanley E. (2017b). Short-form mindfulness training protects against working memory degradation over high-demand intervals. J. Cogni. Enhanc. 1, 154–171. doi: 10.1007/s41465-017-0035-2

[ref57] Johnson D. C. Thom N. J. Stanley E. A. Haase L. Simmons A. N. Shih P. A. B.. (2014). Modifying resilience mechanisms in at-risk individuals: a controlled study of mindfulness training in marines preparing for deployment. Am. J. Psychiatr. 171, 844–853. doi: 10.1176/appi.ajp.2014.13040502, PMID: 24832476 PMC4458258

[ref58] Kahneman D. (1973). Attention and Effort. Vol. 1063. Englewood Cliffs, NJ: Prentice-Hall Inc.

[ref59] Kavanagh J.. (2005). Stress and Performance A Review of the Literature and its Applicability to the Military. Available at: https://www.rand.org/pubs/technical_reports/TR192.html (Accessed June 20, 2020).

[ref60] Keinan G. Friedland N. Sarig-Naor V. (1990). Training for task performance under stress: the effectiveness of phased training methods. J. Appl. Soc. Psychol. 20, 1514–1529. doi: 10.1111/j.1559-1816.1990.tb01490.x

[ref61] Kerick S. E. Allender L. E. (2004). “Effects of cognitive workload on decision accuracy, shooting performance, and cortical activity of soldiers transformational science and Technology for the Current and Future Force.” in *Proceedings of the 24th US Army Science Conference*; November 29–December 2, 2006, 359-362.

[ref62] Kohn H. (1954). The effect of variations of intensity of experimentally induced stress situations upon certain aspects of perception and performance. J. Genet. Psychol. 85, 289–304. doi: 10.1080/00221325.1954.10532884, PMID: 13221791

[ref63] Kreuz M. E. Rose R. M. Jennings C. (1972). Suppression of plasma testosterone levels and psychological stress: a longitudinal study of young men in officer candidate school. Arch. Gen. Psychiatry 26, 479–482. doi: 10.1001/archpsyc.1972.01750230089017, PMID: 5019887

[ref64] Lazarus R. S. (2000). “Evolution of a model of stress, coping, and discrete emotions,” in Handbook of Stress, Coping, and Health: Implications for Nursing Research, Theory, and Practice. 2nd *Edn*. ed. Rice V. H. (Thousand Oaks, CA: SAGE Publications Ltd), 195–222.

[ref65] Lazarus R. S. (2007). “Stress and emotion: A new synthesis,” in The Praeger Handbook on Stress and Coping. Vol 1. eds. Monat A. Lazarus R. S. Reevy G. (Westport, Connecticut: Praeger), 33–51.

[ref66] Lazarus R. S. Folkman S. (1984). Stress, Appraisal, and Coping. New York, NY: Springer.

[ref67] Lazarus R. S. Folkman S. (1987). Transactional theory and research on emotions and coping. Eur. J. Personal. 1, 141–169. doi: 10.1002/per.2410010304

[ref68] Lieberman H. R. Bathalon G. P. Falco C. M. Kramer F. M. Morgan C. A. Niro P. (2005a). Severe decrements in cognition function and mood induced by sleep loss, heat, dehydration, and undernutrition during simulated combat. Biol. Psychiatry 57, 422–429. doi: 10.1016/j.biopsych.2004.11.014, PMID: 15705359

[ref69] Lieberman H. R. Bathalon G. P. Falco C. M. Morgan C. A. Niro P. J. Tharion W. J. (2005b). The fog of war: decrements in cognitive performance and mood associated with combat-like stress. Aviat. Space Environ. Med. 76, C7–C14. 16018323

[ref70] Lieberman H. R. Farina E. K. Caldwell J. Williams K. W. Thompson L. A. Niro P. J.. (2016). Cognitive function, stress hormones, heart rate and nutritional status during simulated captivity in military survival training. Physiol. Behav. 165, 86–97. doi: 10.1016/j.physbeh.2016.06.037, PMID: 27374427

[ref71] Lieberman H. R. Tharion W. J. Shukitt-Hale B. Speckman K. L. Tulley R. (2002). Effects of caffeine, sleep loss, and stress on cognitive performance and mood during US navy SEAL training. Psychopharmacology 164, 250–261. doi: 10.1007/s00213-002-1217-9, PMID: 12424548

[ref72] Lukey B. J. Tepe V. (eds.) (2008). Biobehavioral Resilience to Stress. Boca Raton, FL: CRC Press.

[ref73] Luthar S. S. Cicchetti D. (2000). The construct of resilience: implications for interventions and social policies. Dev. Psychopathol. 12, 857–885. doi: 10.1017/S0954579400004156, PMID: 11202047 PMC1903337

[ref74] Martin K. McLeod E. Périard J. Rattray B. Keegan R. Pyne D. B. (2019). The impact of environmental stress on cognitive performance: a systematic review. Hum. Factors 61, 1205–1246. doi: 10.1177/0018720819839817, PMID: 31002273

[ref75] Martin K. Périard J. Rattray B. Pyne D. B. (2020). Physiological factors which influence cognitive performance in military personnel. Hum. Factors 62, 93–123. doi: 10.1177/0018720819841757, PMID: 31009241

[ref76] Martin K. Thompson K. G. Keegan R. Ball N. Rattray B. (2015). Mental fatigue does not affect maximal anaerobic exercise performance. Eur. J. Appl. Physiol. 115, 715–725. doi: 10.1007/s00421-014-3052-1 25425259

[ref77] Mastroianni G. R. Mabry T. R. Benedek D. M. Ursano R. J. (2008). “The stresses of modern war,” in Biobehavioral Resilience to Stress. eds. Lukey B. J. Tepe V. (Boca Raton, FL: CRC Press), 50–62.

[ref78] Meichenbaum D. (1977). Cognitive-Behaviour Modification: An Integrative Approach. New York: Springer.

[ref79] Mikulincer M. Florian V. (1995). Appraisal of and coping with a real-life stressful situation: the contribution of attachment styles. Personal. Soc. Psychol. Bull. 21, 406–414. doi: 10.1177/0146167295214011

[ref80] Morgan C. A. Doran A. Steffian G. Hazlett G. Southwick S. M. (2006). Stress-induced deficits in working memory and visuo-constructive abilities in special operations soldiers. Biol. Psychiatry 60, 722–729. doi: 10.1016/j.biopsych.2006.04.021, PMID: 16934776

[ref81] Morgan C. A. Rasmusson A. M. Wang S. Hoyt G. Hauger R. L. Hazlett G. (2002). Neuropeptide-Y, cortisol, and subjective distress in humans exposed to acute stress: replication and extension of previous report. Biol. Psychiatry 52, 136–142. doi: 10.1016/S0006-3223(02)01319-7, PMID: 12114005

[ref82] Morgan C. A. Russell B. McNeil J. Maxwell J. Snyder P. J. Southwick S. M.. (2011). Baseline burnout symptoms predict Visuospatial executive function During survival school training in special operations military personnel. J. Int. Neuropsychol. Soc. 17, 494–501. doi: 10.1017/S1355617711000221, PMID: 21466738

[ref83] Morgan C. A. Wang S. Mason J. Southwick S. M. Fox P. Hazlett G.. (2000). Hormone profiles in humans experiencing military survival training. Biol. Psychiatry 47, 891–901. doi: 10.1016/S0006-3223(99)00307-8, PMID: 10807962

[ref84] Mujica-Parodi L. R. Renelique R. Taylor M. K. (2008). Higher body fat percentage is associated with increased cortisol reactivity and impaired cognitive resilience in response to acute emotional stress. Int. J. Obes. 33, 157–165. doi: 10.1038/ijo.2008.218, PMID: 19015661

[ref85] Negash S. Xie S. Davatzikos C. Clark C. M. Trojanowski J. Q. Shaw L. M.. (2013). Cognitive and functional resilience despite molecular evidence of Alzheimer’s disease pathology. Alzheimers Dement. 9, e89–e95. doi: 10.1016/j.jalz.2012.01.009, PMID: 23127468 PMC3640705

[ref86] Nibbeling N. Oudejans R. R. Ubink E. M. Daanen H. A. (2014). The effects of anxiety and exercise-induced fatigue on shooting accuracy and cognitive performance in infantry soldiers. Ergonomics 57, 1366–1379. doi: 10.1080/00140139.2014.924572, PMID: 24926568

[ref87] Nicholls A. R. Levy A. R. Carson F. Thompson M. A. Perry J. L. (2016). The applicability of self-regulation theories in sport: goal adjustment capacities, stress appraisals, coping, and well-being among athletes. Psychol. Sport Exerc. 27, 47–55. doi: 10.1016/j.psychsport.2016.07.011

[ref88] Nicholls A. R. Perry J. L. Calmeiro L. (2014). Precompetitive achievement goals, stress appraisals, emotions, and coping among athletes. J. Sport Exerc. Psychol. 36, 433–445. doi: 10.1123/jsep.2013-0266, PMID: 25356608

[ref89] Nindl B. C. Billing D. C. Drain J. R. Beckner M. E. Greeves J. Groeller H.. (2018). Perspectives on resilience on military readiness and preparedness: report of an international military physiology roundtable. J. Sci. Med. 21, 1116–1124. doi: 10.1016/j.jsams.2018.05.005, PMID: 29886134

[ref90] Orantes-Gonzalez E. Heredia-Jimenez J. (2021). Obstacle course soldiers’ reaction and perception time: combat equipment load effects. J. Psychol. Afr. 31, 434–438. doi: 10.1080/14330237.2021.1952731

[ref91] Pallavicini F. Argenton L. Toniazzi N. Aceti L. Mantovani F. (2016). Virtual reality applications for stress management training in the military. Aerospace Med. Human Perfor. 87, 1021–1030. doi: 10.3357/AMHP.4596.2016, PMID: 28323588

[ref92] Parkes K. R. (1986). Coping in stressful episodes: The role of individual differences, environmental factors, and situational characteristics. J. Pers. Soc. Psychol. 51, 1277–1292. doi: 10.1037/0022-3514.51.6.1277, PMID: 3806363

[ref93] Parsons T. D. Rizzo A. A. (2008). Initial validation of a virtual environment for assessment of memory functioning: virtual reality cognitive performance assessment test. Cyberpsychol. Behav. 11, 17–25. doi: 10.1089/cpb.2007.9934, PMID: 18275308

[ref94] Pflanz S. (2001). Occupational stress and psychiatric illness in the military: investigation of the relationship between occupational stress and mental illness among military mental health patients. Mil. Med. 166, 457–462. doi: 10.1093/milmed/166.6.457, PMID: 11413719

[ref95] Pflanz S. E. Ogle A. D. (2006). Job stress, depression, work performance, and perceptions of supervisors in military personnel. Mil. Med. 171, 861–865. doi: 10.7205/MILMED.171.9.861, PMID: 17036607

[ref96] Pflanz S. Sonnek S. (2002). Work stress in the military: prevalence, causes, and relationship to emotional health. Mil. Med. 167, 877–882. doi: 10.1093/milmed/167.11.877, PMID: 12448610

[ref97] Rabheru K. Cassidy K.-L. Cassidy B. Conn D. (2021). “Mental Health, Cognitive Resilience, and Vitality,” in Promoting the Health of Older Adults: The Canadian Experience. eds. Rootman I. Edwards P. Levasseur M. Grunberg F. (Canada: Canadian Scholars’ Press), 323.

[ref98] Rezapour T. Assari S. Kirlic N. Vassileva J. Ekhtiari H. (2021). Enhancing Cognitive Resilience in Adolescence and Young Adults: A Multidimensional Approach Family Resilience and Recovery from Opioids and Other Addictions. Cham: Springer, 45–64.

[ref99] Rizzo A. John B. Newman B. Williams J. Hartholt A. Lethin C.. (2013). Virtual reality as a tool for delivering PTSD exposure therapy and stress resilience training. Mil. Behav. Health 1, 52–58. doi: 10.1080/21635781.2012.721064

[ref100] Rizzo A. Parsons T. D. Lange B. Kenny P. Buckwalter J. G. Rothbaum B.. (2011). Virtual reality goes to war: a brief review of the future of military behavioral healthcare. J. Clin. Psychol. Med. Settings 18, 176–187. doi: 10.1007/s10880-011-9247-2, PMID: 21553133

[ref101] Roesch S. C. Weiner B. Vaughn A. A. (2002). Cognitive approaches to stress and coping. Curr. Opin. Psychiatry 15, 627–632. doi: 10.1097/00001504-200211000-00012

[ref102] Rossetto K. R. (2015). Developing conceptual definitions and theoretical models of coping in military families during deployment. J. Fam. Commun. 15, 249–268. doi: 10.1080/15267431.2015.1043737

[ref103] Roth S. Cohen L. J. (1986). Approach, avoidance, and coping with stress. Am. Psychol. 41, 813–819. doi: 10.1037/0003-066X.41.7.813 3740641

[ref104] Rutter M. (1987). Psychosocial resilience and protective mechanisms. Am. J. Orthopsychiatry 57, 316–331. doi: 10.1111/j.1939-0025.1987.tb03541.x 3303954

[ref105] Sandi C. (2013). Stress and cognition. Wiley Interdiscip. Rev. Cogn. Sci. 4, 245–261. doi: 10.1002/wcs.1222 26304203

[ref106] Seitz C. A. Poyrazli S. Harrisson M. A. Flickinger T. Turkson M. (2014). Virtual reality exposure therapy for military veterans with posttraumatic stress disorder: a systematic review. New Sch. Psychol. Bull. 11, 15–29.

[ref107] Sharma V. M. Sridharan K. Selvamurthy W. Mukherjee A. K. Kumaria M. M. L. Upadhyay T. N.. (1994). Personality traits and performance of military parachutist trainees. Ergonomics 37, 1145–1155. doi: 10.1080/00140139408964894, PMID: 8050403

[ref108] Shields M. R. Brooks M. A. Koltyn K. F. Kim J.-S. Cook D. B. (2017). Cognitive resilience and psychological responses across a collegiate rowing season. Med. Sci. Sports Exerc. 49, 2276–2285. doi: 10.1249/mss.0000000000001363, PMID: 28682806

[ref109] Skinner E. A. Edge K. Altman J. Sherwood H. (2003). Searching for the structure of coping: a review and critique of category systems for classifying ways of coping. Psychol. Bull. 129, 216–269. doi: 10.1037/0033-2909.129.2.216, PMID: 12696840

[ref110] Spivak T. Hollands J. G. Kramkowski E. W. (2019). “Cognitive load and situation awareness for soldiers: using an auditory detection response task.” in *Proceedings of the Human Factors and Ergonomics Society Annual Meeting*, October 28, 2019. Sage CA: Los Angeles, CA: SAGE Publications. 10.1177/0018720819825803 30768371

[ref111] Staal M. A. (2004). Stress, Cognition, and Human Performance: A Literature Review and Conceptual Framework. Available at: https://ntrs.nasa.gov/search.jsp?R=20060017835 (Accessed June 20, 2020).

[ref112] Staal M. A. Bolton A. Yaroush R. Bourne L. (2008). “Cognitive performance and resilience to stress,” in Biobehavioral Resilience to Stress. eds. Lukey B. J. Tepe V. (Boca Raton, FL: CRC Press), 259–299.

[ref113] Stefanopoulou E. Hirsch C. R. Hayes S. Adlam A. Coker S. (2014). Are attentional control resources reduced by worry in generalized anxiety disorder? J. Abnorm. Psychol. 123, 330–335. doi: 10.1037/a0036343, PMID: 24886007 PMC4067229

[ref114] Taverniers J. Smeets T. Lo Bue S. Syroit J. Van Ruysseveldt J. Pattyn N.. (2011). Visuo-spatial path learning, stress, and cortisol secretion following military cadets’ first parachute jump: the effect of increasing task complexity. Cogn. Affect. Behav. Neurosci. 11, 332–343. doi: 10.3758/s13415-011-0043-0, PMID: 21607782

[ref115] Taverniers J. Taylor M. K. Smeets T. (2013). Delayed memory effects after intense stress in special forces candidates: exploring path processes between cortisol secretion and memory recall. Stress: The international journal on the biology of. Stress 16, 311–320. doi: 10.3109/10253890.2012.721824, PMID: 22900536

[ref116] Taverniers J. Van Ruysseveldt J. Smeets T. von Grumbkow J. (2010). High-intensity stress elicits robust cortisol increases, and impairs working memory and visuo-spatial declarative memory in special forces candidates: a field experiment. Stress 13, 324–334. doi: 10.3109/10253891003642394, PMID: 20536334

[ref117] Taylor A. H. Schatz S. (2011). “Measuring the Effectiveness of Stress Prevention Programs in Military Personnel.” in *Paper presented at the International Conference on Foundations of Augmented Cognition*, July 9, 2011. Berlin, Heidelberg.

[ref118] Vrijkotte S. Roelands B. Meeusen R. Pattyn N. (2016). Sustained military operations and cognitive performance. Aerospace Med. Human Perform. 87, 718–727. doi: 10.3357/AMHP.4468.2016, PMID: 27634607

[ref119] Wiederhold B. K. Wiederhold M. D. (2008). Virtual reality for posttraumatic stress disorder and stress inoculation training. J. Cyber Ther. Rehabil. 1, 23–35.

[ref120] Yaffe K. Fiocco A. J. Lindquist K. Vittinghoff E. Simonsick E. M. Newman A. B.. (2009). Predictors of maintaining cognitive function in older adults: the health ABC study. Neurology 72, 2029–2035. doi: 10.1212/WNL.0b013e3181a92c36, PMID: 19506226 PMC2692177

[ref121] Zeidan F. Johnson S. K. Diamond B. J. David Z. Goolkasian P. (2010). Mindfulness meditation improves cognition: evidence of brief mental training. Conscious. Cogn. 19, 597–605. doi: 10.1016/j.concog.2010.03.014, PMID: 20363650

